# Ranibizumab treatment patterns in prior ranibizumab-treated neovascular age-related macular degeneration patients: Real-world outcomes from the LUMINOUS study

**DOI:** 10.1371/journal.pone.0244183

**Published:** 2020-12-30

**Authors:** Frank G. Holz, Angelo M. Minnella, Raman Tuli, Pradeepa Yoganathan, Soumil Parikh, Robin Hamilton, on behalf of the LUMINOUS study group

**Affiliations:** 1 Department of Ophthalmology, University of Bonn, Bonn, Germany; 2 Department of Ophthalmology, Catholic University of Sacred Heart—Foundation “Policlinico Universitario A. Gemelli"—IRCCS, Rome, Italy; 3 The Retina Centre of Ottawa, Ottawa, Canada; 4 Department of Ophthalmology, University of Ottawa, Ottawa, Canada; 5 Department of Ophthalmology and Vision Sciences, University of Toronto, Toronto, Ontario, Canada; 6 Windsor Eye Associates, Windsor, Ontario, Canada; 7 Kresge Eye Institute, Wayne State University, Detroit, Michigan, United States of America; 8 Novartis Pharma AG, Basel, Switzerland; 9 Department of Medical Retina, Moorfields Eye Hospital NHS Foundation Trust and National Institute for Health Research (NIHR) Biomedical Research Centre (BRC) at Moorfields Eye Hospital, London, United Kingdom; Massachusetts Eye & Ear Infirmary, Harvard Medical School, UNITED STATES

## Abstract

**Purpose:**

To evaluate the effectiveness, safety, and treatment patterns of ranibizumab 0.5 mg in prior ranibizumab-treated patients with neovascular age-related macular degeneration (nAMD) enrolled in the LUMINOUS^™^ study.

**Patients and methods:**

LUMINOUS, a 5-year, prospective, multicenter, observational study, recruited 30,138 adult patients (treatment-naïve or prior ranibizumab-treated or other ocular treatments) across all approved indications for ranibizumab. Patients were treated as per local ranibizumab label of participating countries. Here we report the mean change in visual acuity (VA) at Year 1, treatment exposure, overall incidence of ocular, non-ocular adverse events (AEs) and serious AEs (SAEs) in prior ranibizumab-treated nAMD patients (n = 16,167).

**Results:**

At baseline, the mean (standard deviation [SD]) age of patients was 78.4 (9.0) years, 59.0% were female, and 80.0% were Caucasian. At Year 1 (n = 10,168), the mean (SD) VA change was −1.6 (12.6) letters (baseline VA: 58.3 [19.0] letters) with a mean (SD) of 4.7 (3.1) ranibizumab injections. Stratified by duration of prior ranibizumab treatment of <1 (n = 4,112), 1 to <2 (n = 2,095), 2 to <3 (n = 1,506), 3 to <4 (n = 1,123), 4 to <5 (n = 689), and ≥5 (n = 256) years, the mean (SD) VA change at Year 1 were −1.2 (13.5), −2.0 (12.3), −2.0 (11.3), −1.9 (11.8), −2.5 (10.9), and 0.0 (11.2) letters, respectively. Mean (SD) VA change in patients who received ≤6 and >6 injections over 1 year was −1.8 (13.8) and +0.5 (12.5) letters, respectively. The rate of ocular/non-ocular AEs and SAEs across all prior ranibizumab-treated patients over 5 years were 13.29%/23.02% and 0.84%/13.66%, respectively.

**Conclusions:**

Overall, regardless of the prior ranibizumab-treatment duration, VA was maintained in these patients at Year 1, and those receiving ≥6 injections showed a trend towards gaining letters. There were no new safety signals. These results may help inform routine clinical practice to appropriately treat nAMD patients with ranibizumab to achieve optimal visual outcomes.

## Introduction

Neovascular age-related macular degeneration (nAMD) is one of the leading causes of vision impairment in the elderly population [1–3]. The global burden of the disease is projected at 196 million patients by 2020 [4].

Over the last couple of decades, various treatment options for nAMD have evolved with the use of laser, steroid, verteporfin photodynamic therapy, and in the last decade, the vascular endothelial growth factors inhibitors (anti-VEGF) are considered as a breakthrough in therapy. Anti-VEGFs are currently the treatment of choice for nAMD as well as for neovascularization and macular edema due to other causes [5].

A wealth of scientific evidence from randomized clinical trials (RCTs) and extension studies have proven the efficacy and safety of ranibizumab in the treatment of nAMD [6–9]. It has been over a decade since ranibizumab was approved for the treatment of patients with nAMD in the European Union, the United States (US) and many other countries worldwide, after observing robust visual gains in the two Phase III studies, ANCHOR and MARINA [6, 8]. Over time, ranibizumab therapy for nAMD has also evolved from monthly injections, to a *pro re nata* (PRN) regimen and a Treat and Extend (T&E) approach, aiming to give a more individualized treatment option to patients. Studies from Europe, Australia, and the US have shown a 46–72% decrease in the incidence of blindness since the use of ranibizumab for the treatment of patients with nAMD [10–14].

Data from RCTs and about 5.5 million patient-years of experience demonstrates the robust efficacy and safety profile of ranibizumab across all approved indications [15, 16]. Although there is ample evidence for ranibizumab for treatment outcomes from RCTs, it is important to understand the effectiveness of ranibizumab when translated to the real-world setting. Real-world evidence (RWE) helps us elucidate ranibizumab treatment patterns, treatment exposure, effectiveness in a heterogeneous population, patient access to treatment, and patient management. The RWE studies also help understand the challenges from the patients’ perspective to identifying the right treatment option [17]. Thus, the information from both RCTs and RWE studies may help the physician to optimize the evidence-based treatment for patients.

LUMINOUS (NCT01318941), the largest prospective, observational, multicenter study in medical retina was designed to evaluate the effectiveness and safety of ranibizumab in broader patient populations in real-world clinical practice [18]. This study enrolled patients (N = 30,138) who were treatment-naïve, prior ranibizumab-treated, and prior-treated with other ocular treatments across all the approved indications for ranibizumab. While treatment-naïve patients with nAMD from the LUMINOUS study have demonstrated gains in visual acuity (VA) at 1-year, it is of interest to evaluate the effectiveness and safety of continued ranibizumab treatment in nAMD patients who had already received ranibizumab therapy before entering the study [18]. The 1-year visual acuity (VA), treatment patterns, and long-term safety outcomes of prior ranibizumab-treated patients with nAMD from the LUMINOUS study are reported here.

## Materials and methods

### Study design

LUMINOUS was a 5-year (21 March 2011 to 27 April 2016), prospective, observational, multicenter, open-label, single-arm, global study conducted at 488 clinical sites across 42 countries (NCT01318941). The study design is described elsewhere [18].

The study was conducted in accordance with the Guidelines for Good Pharmacoepidemiology Practices issued by the International Society for Pharmacoepidemiology, with any applicable national guidelines, and ethical principles laid down in the Declaration of Helsinki. The study protocol was reviewed and approved by an Independent Ethics Committee or Institutional Review Board for each center (S1 Table). All participants provided written informed consent.

### Participants and treatment

Consenting adult patients, who were either treatment-naïve, or prior-treated with ranibizumab or another ocular therapy for any of the approved indications included in the local product label of the participating countries, were enrolled. Patients were excluded if they were participating in other investigational studies or if they had received systemic anti-VEGF therapy in 90 days or ocular anti-VEGF therapy other than ranibizumab 30 days prior to enrollment. Eyes previously treated with ranibizumab (prior ranibizumab-treated/-prior other ocular-treated) were defined as eyes that have been pre-treated with at least one treatment of ranibizumab regardless of other treatments. Patients exposed to both ranibizumab and other ocular treatments in the primary treated eye prior to baseline are included in the prior ranibizumab-treated group only.

Enrolled patients were treated with intravitreal ranibizumab 0.5 mg as per the local product label and local clinical practices. Further retreatments were performed at investigator’s discretion. The first eye treated during the study was considered the primary treated eye. If both eyes were first treated on the same date, or if both eyes were pre-treated, the eye with the earliest diagnosis date was considered the primary treated eye. Since the patients were recruited over time, and the calendar time point of study completion was pre-set, the review (follow-up) period varied according to entry dates. The minimum potential follow-up for each patient was defined as 1 year in the protocol. Visits took place at a frequency determined by the investigator. Data from all visits were documented in the electronic case report form (eCRF). It was recommended to capture data in the eCRF at every visit or at a minimum of every 3 months. Investigators were encouraged to follow-up with patients who were not seen in the clinic for at least 6 months since the last visit, in order to capture data. Patients not seen at least once per year, or those switched to another anti-VEGF therapy, were discontinued from the study.

Information regarding pre-treatment with ranibizumab or other intravitreal medications was recorded on the prior and concomitant medications/significant non-drug therapies eCRF page. For each patient, only data collected over the 365 days after the baseline date (±45 days; Day 319–409) were included. The time periods were not mutually exclusive, so patients might have visited at different time periods simultaneously. If so, these patients were classified as lost to follow-up in this study.

### Assessments

Demographic and baseline characteristics were collected at study start (baseline), including ocular and non-ocular medical history, primary indication for initiation of ranibizumab treatment, and prior ocular treatments/therapies. These are presented using standard descriptive statistics and by pre-treatment status, indication, and time period.

Effectiveness assessments included VA (preferably best-corrected VA) evaluation by each participating investigator as a part of routine care practice using Early Treatment Diabetic Retinopathy Study (ETDRS) letters, or Snellen charts or equivalent. To facilitate data analysis, Snellen fractions and decimals were converted to the ETDRS equivalent letter scores. It was recommended that the same method of VA assessment be used throughout the study wherever possible. All AEs, including SAEs, irrespective of suspected causal association that occurred during the study were collected.

Overall, the number of ranibizumab injections administered over time, the average time interval (in weeks) between consecutive injections, visit frequency, and treatment patterns (unilateral [involving single eye]/bilateral [involving both eyes]) were recorded. The proportion of patients receiving ocular and non-ocular concomitant medications was also documented.

### Statistical analysis

Due to the design of the study, 1-year data were potentially available for all patients, while the availability of data for subsequent years was dependent on the patient’s study entry date. The effectiveness data are therefore presented here for the time period up to 1 year.

All effectiveness and safety data were summarized descriptively. The enrolled set included all patients who signed informed consent and had at least the baseline assessment. The safety set comprised patients in the enrolled set who were treated with at least one dose of ranibizumab during the study or prior to the start of the study and had at least one safety assessment after the first treatment. The primary treated eye set included all primary treated eyes in patients from the safety set and was the primary analysis set for effectiveness.

The baseline date for the primary treated eye was the date of study entry if the primary treated eye had been pre-treated with ranibizumab (Study Day 1). The primary effectiveness variable was the mean change in VA ETDRS letter score from baseline presented by quarterly and yearly periods for the primary treated eye set. Effectiveness data are presented for patients in the primary treated eye set who provided baseline and Year 1 data. The mean change in VA from baseline at Year 1 was presented by injection frequency during Year 1 (≤6, >6), and the duration of prior ranibizumab treatment. Further VA evaluations include the proportions of patients with a VA loss (defined as ≤0 letter change from baseline) or gain (defined as >0 letter change from baseline) of >0 to <5 letters, 5 to <10 letters, 10 to <15 letters, and ≥15 letters at Year 1.

The number of injections and monitoring visits up to 1 year were summarized for patients with participation for at least 365 days in the study. Safety was assessed based on the incidence proportion, relationship, and severity of treatment-emergent ocular and non-ocular AEs. Ocular AEs were assessed for the primary treated eye set and non-ocular AEs were assessed for the safety set over 5 years.

## Results

### Study enrollment, patient demographics, and baseline ocular characteristics

**Fig 1:**
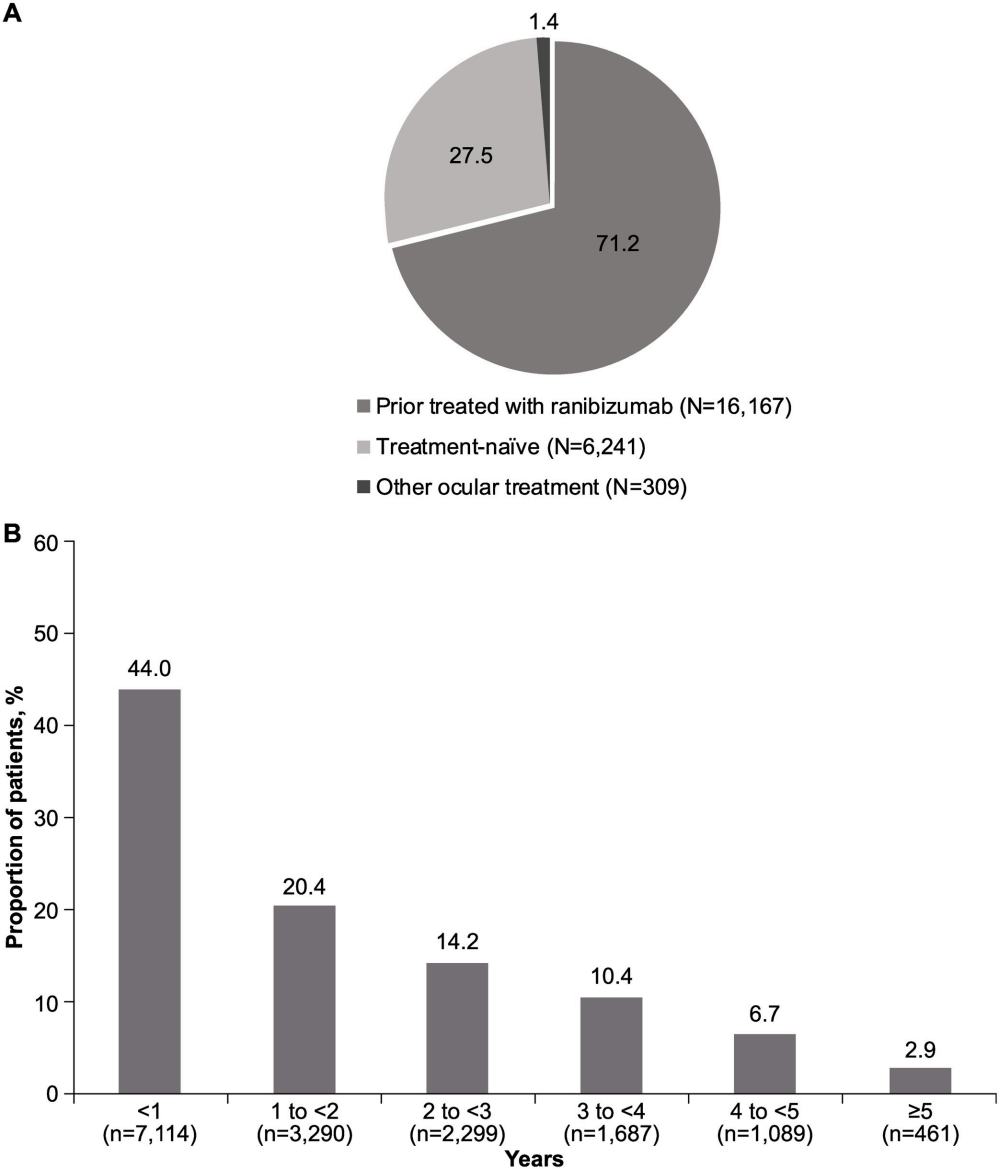
**A.** Proportion of nAMD patients by treatment status group (%). N, total number of patients; nAMD, neovascular age-related macular degeneration. **B.** Duration of prior ranibizumab treatment (N = 16,167). N, total number of patients; n, number of patients; nAMD, neovascular age-related macular degeneration.

The LUMINOUS study enrolled a total of 30,138 patients across all approved ranibizumab indications (nAMD, diabetic macular edema, branch retinal vein occlusion, central retinal vein occlusion, and myopic choroidal neovascularization) worldwide. Overall, 75.4% (n = 22,717) of patients in the safety set had nAMD, of whom 16,167 (71.2%) were prior ranibizumab-treated patients (Fig 1A). Amongst the prior ranibizumab-treated patients, 7,114 (44%) patients had received prior ranibizumab treatment for <1 year (Fig 1B).

At the end of Year 1, 12,629 prior ranibizumab-treated patients with nAMD remained in the LUMINOUS study. The most frequent reasons for study discontinuation were switch to another anti-VEGF (9.5%; n = 1,541) and loss to follow-up (5.6%, n = 898). As per the study design, visits were scheduled at the discretion of the investigator and could fall outside the 12-month window. Hence, patients may have continued in the study but not been included in the analysis if they did not have any visits in the 1-year window (after the baseline date [±45 days; Day 319 to Day 409]). In the primary treated eye set, baseline and 1-year VA data were available for 10,168 prior ranibizumab-treated patients with nAMD. The safety set included 16,167 prior ranibizumab-treated patients with nAMD.

At baseline, the mean (standard deviation [SD]) age of patients was 78.4 (9.0) years, most were Caucasian (79.9%), and the majority were female (59.0%) (Table 1). Patients had a broad range of comorbidities with hypertension (60.1%), hypercholesterolemia (34.5%), and diabetes (15.5%) being the most common. Ocular concomitant medications and significant non-drug therapies were reported for 28.5% of patients in the primary treated eye set, and non-ocular concomitant medications and significant non-drug therapies were reported for 69.5% of patients in the safety set.

**Table 1:** Baseline demographics and ocular characteristics for prior ranibizumab-treated patients with nAMD.

Characteristics	Prior ranibizumab-treated patients with nAMD N = 16,167*
**Patient demographics**	
Mean (SD) age, years	78.4 (9.0)
Gender, n (%)	
Male	6637 (41.1)
Female	9530 (59.0)
Race, n (%)	
Caucasian	12,915 (79.9)
Asian	2427 (15.0)
Native American	11 (0.1)
Pacific Islander	1 (0.01)
Black	42 (0.3)
Other	374 (2.3)
**Ocular characteristics**	
VA	
n^†^	15,165
Mean (SD) VA, ETDRS letters	56.5 (20.0)
CRT	
n^†^	11,639
Mean (SD) CRT, μm	279.4 (106.0)
IOP	
n^†^	7911
Mean (SD), mmHg	15.3 (3.6)
Median time from diagnosis to first treatment, days	557.0

Safety set.

* number of patients at enrollment;

^†^number of evaluable baseline patients.

For prior ranibizumab-treated eyes, the date of study entry was considered the baseline date.

Time since diagnosis = time between diagnosis and study entry for primary treated eyes pre-treated with ranibizumab and time between diagnosis and date of first on-study injection with ranibizumab if the primary treated eye has not been pre-treated with ranibizumab.

Patients with a baseline visit date present are included. Data collected until the last recorded follow-up date was used to perform the analyses (i.e. data for 5-year duration of the study).

N, total number of patients; n, number of patients; CRT, central retinal thickness; ETDRS, Early Treatment Diabetic Retinopathy Study; IOP, intraocular pressure; nAMD, neovascular age-related macular degeneration; SD, standard deviation; VA, visual acuity.

**Fig 2:**
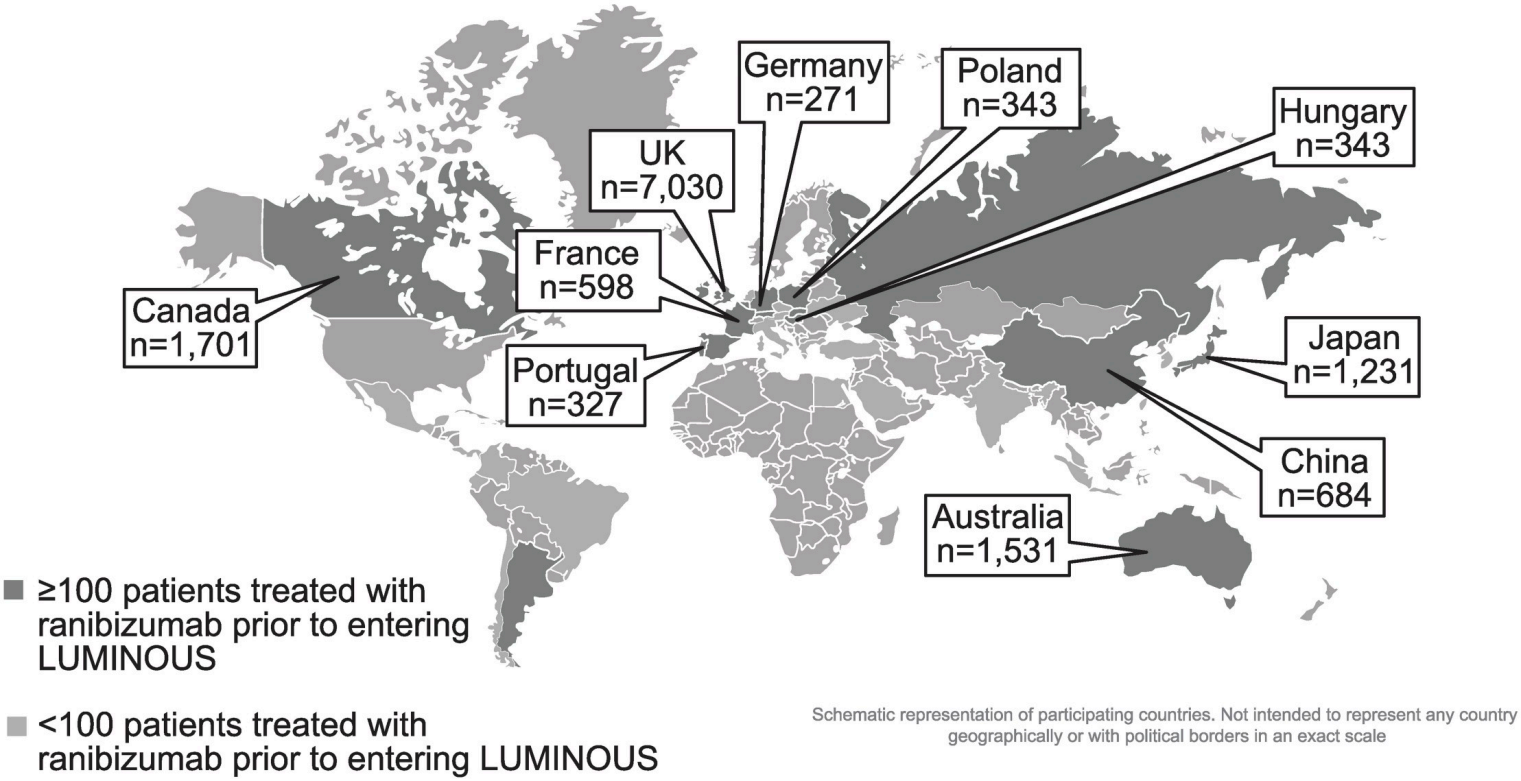
Country-specific enrollment of prior ranibizumab-treated patients with nAMD. Pop-out boxes only displayed for top 10 countries with nAMD patients treated with ranibizumab prior to entering LUMINOUS. n, number of patients; nAMD, neovascular age-related macular degeneration; UK, United Kingdom.

Approximately 87% of prior ranibizumab-treated patients with nAMD were recruited from 10 countries–the UK (43.5%), Canada (10.5%), Australia (9.5%), Japan (7.6%), China (4.2%), Poland (2.1%), Germany (1.7%), France (3.7%), Hungary (2.1%), and Portugal (2.0%; Fig 2).

### Efficacy outcomes

**Fig 3:**
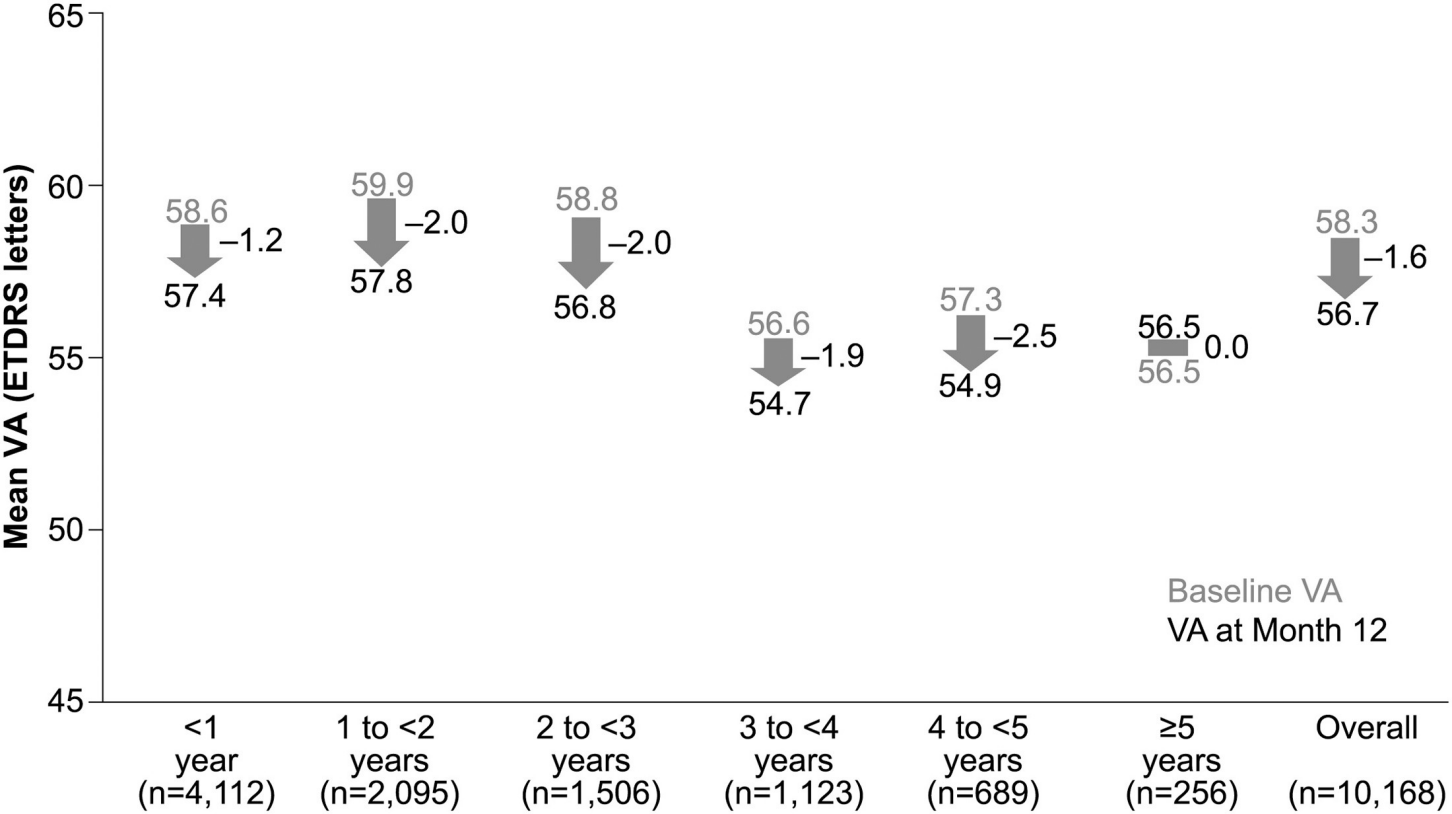
VA outcomes at Year 1 stratified by duration of treatment. Primary treated eye set; n, number of patients with evaluable patients with baseline and Month 12 data; ETDRS, Early Treatment Diabetic Retinopathy Study; VA, visual acuity.

The mean (SD) VA change in letters at Year 1 for 10,168 patients was −1.6 (12.6) letters from a baseline VA of 58.3 (19.0). Overall, vision was maintained at the end of Year 1 in prior ranibizumab-treated patients. The mean (SD) change in VA at Year 1 was −1.2 (13.5) from a baseline VA of 58.6 (18.5) in patients who received prior ranibizumab treatment for less than 1 year. The final VA achieved by these patients at Year 1 was 57.4 (20.4). When stratified by duration of ranibizumab treatment prior to study entry, the VA change ranged from 0.0 to −2.5 in prior ranibizumab-treated patients who received <1 year to ≥5 years of ranibizumab treatment (Fig 3).

**Fig 4:**
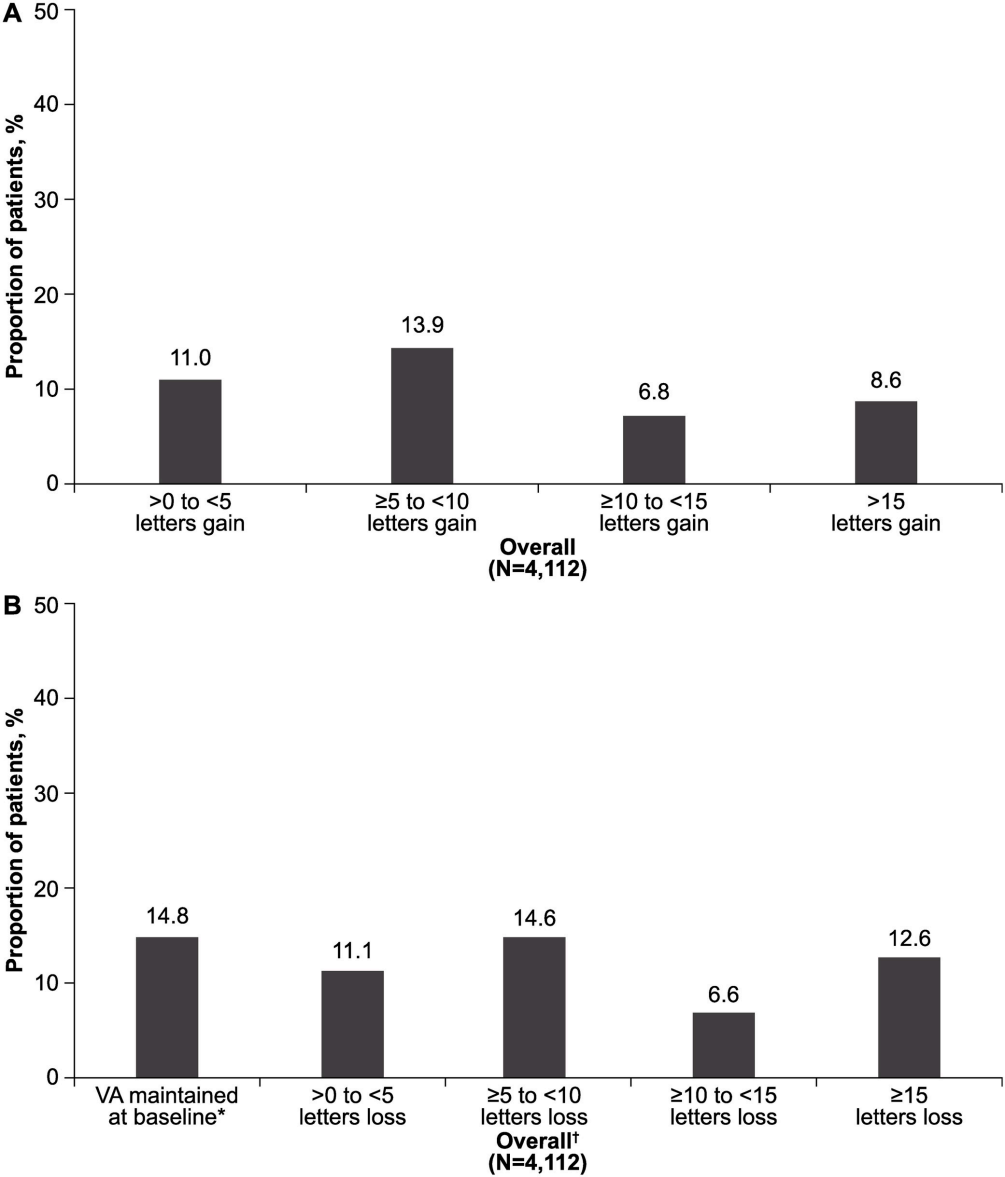
**A.** Overall VA gains at Year 1 in patients who received prior ranibizumab treatment for <1 year. N, number of patients with evaluable baseline and 1-year data; VA, visual acuity. **B.** Overall VA letters lost at Year 1 in patients who received prior ranibizumab treatment for <1 year. *Includes patients with 0 letters loss. ^†^At 1-year, vision was largely maintained in almost half (47.1%) of patients treated with ranibizumab for up to 1 year before entering LUMINOUS. N, number of patients with evaluable baseline and 1-year data; VA, visual acuity.

Around 40.3% (n = 1657) of prior ranibizumab-treated patients gained >0 to ≥15 letters from baseline at Year 1. When further stratified by the number of letters gained, 8.6% (n = 352) gained ≥15 letters (Fig 4A). At Year 1, vision was largely maintained in almost half (47.1%) of patients who were prior ranibizumab-treated for up to 1 year before entering the LUMINOUS study. VA was maintained at baseline levels (0 letter loss) in 14.8% (n = 607) of patients and the proportion of patients with a VA loss of ≥15 letters was 12.6% (n = 520) (Fig 4B).

### Treatment exposure

**Fig 5:**
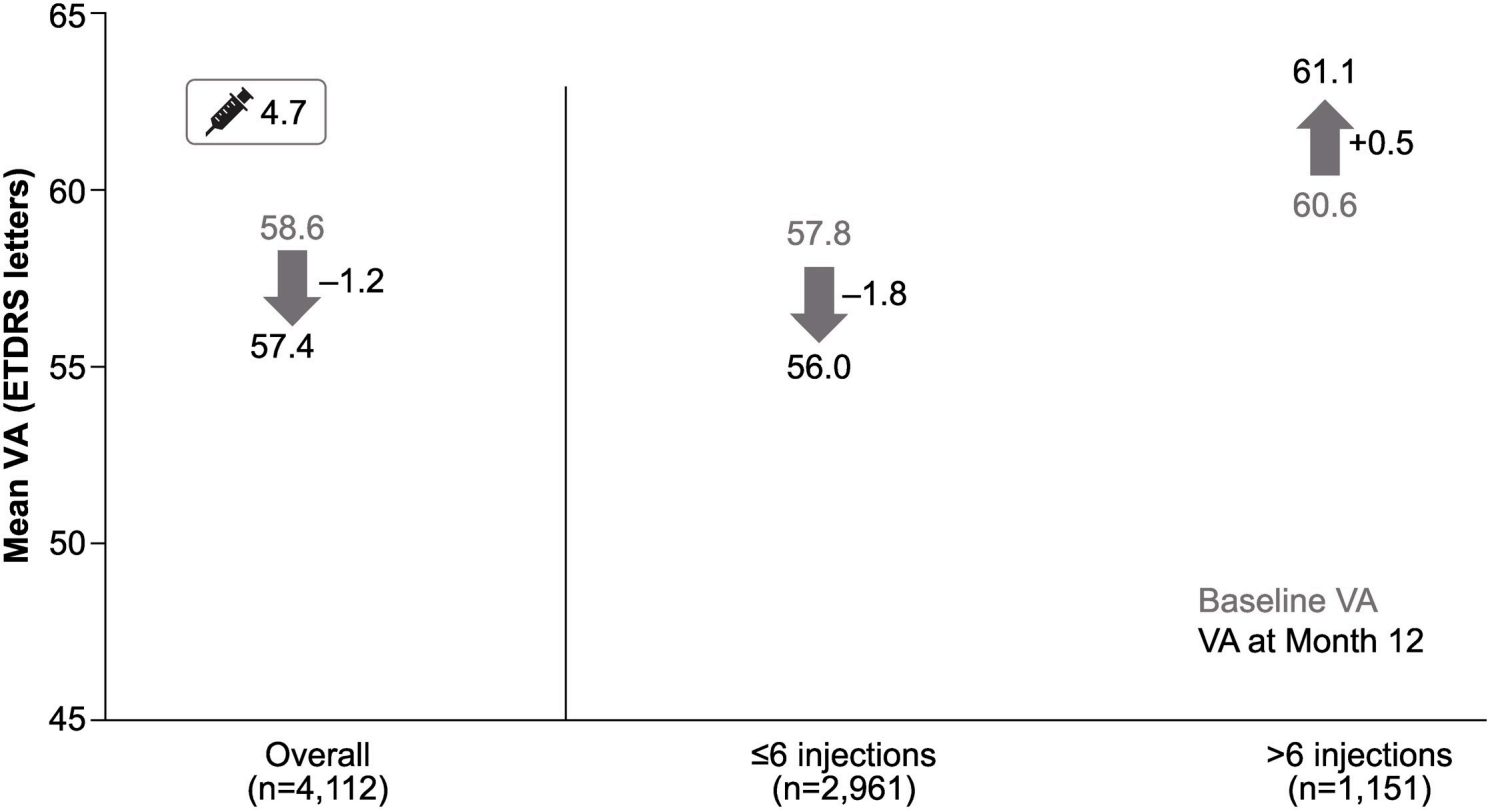
VA outcomes at 1 year in patients who received prior ranibizumab treatment for <1 year: Stratified by injection frequency. n, number of patients with evaluable patients with baseline and Month 12 data; ETDRS, Early Treatment Diabetic Retinopathy Study; VA, visual acuity.

The mean (SD) number of injections over 1 year in prior ranibizumab-treated patients was 4.7 (3.1), and the mean number of monitoring visits was 8.1 (3.8). Overall, 72.0% of patients received ≤6 injections in the first year. In patients with <1 year of ranibizumab treatment before entering the study, a higher number of injections was associated with better VA gains. The mean (SD) change in VA for patients who received ≤6 injections was −1.8 (13.8) from a baseline of 57.8 (19.0), whereas patients who received >6 injections gained 0.5 (12.5) letters from a baseline VA of 60.6 (16.9; Fig 5).

### Country–specific data

The baseline demographics and ocular characteristics are given in S2 Table. Similar to the global cohort, overall, VA was maintained at Year 1 across the ten countries that recruited the most number of prior ranibizumab-treated patients (S3 Table). Among these countries, the baseline VA ranged from 47.6 (China) to 62.7 (Japan) letters, change in VA at Year 1 from −0.1 (Poland) to −4.9 (Portugal) letters, and the VA achieved at Year 1 from 45.4 (China) to 62.0 (Japan) letters. The mean number of ranibizumab injections at Year 1 ranged from 1.9 (Portugal) to 7.5 (in both Australia and Canada; S3 Table).

### Safety outcomes

In prior ranibizumab-treated patients with nAMD (n = 16,167), ocular AEs were reported in 13.29% (n = 2148) of the patients in the primary treated eye set over 5 years. The most common were cataract (2.22%; n = 359), intraocular pressure increase (1.53%, n = 247), and eye pain (1.35%; n = 218; Table 2). The ocular AEs suspected to be related to ranibizumab and/or ocular injection were reported in 4.08% (n = 660) of eyes, of which 1.42% (n = 230) were reported to be related to ranibizumab alone. In all, 8.34% (n = 1349), 4.18% (n = 676), and 0.76% (n = 123) of patients had mild, moderate, and severe ocular AEs, respectively.

**Table 2:** Ocular and non-ocular AEs in prior ranibizumab-treated patients with nAMD.

Prior ranibizumab-treated group (N = 16,167)	
Preferred term	n, (%)
**Ocular AEs**^†^	
Total	2148 (13.29)
Cataract	359 (2.22)
IOP increased	247 (1.53)
Eye pain	218 (1.35)
Dry eye	146 (0.90)
Blepharitis	146 (0.90)
Vitreous floaters	149 (0.92)
Visual acuity reduced	124 (0.77)
Conjunctivitis	97 (0.60)
Conjunctival hemorrhage	93 (0.58)
Posterior capsule opacification	84 (0.52)
**Non-ocular AEs**^†^	
Total	3721 (23.02)
Fall	290 (1.79)
Death	218 (1.35)
Pneumonia	216 (1.34)
Lower respiratory tract infection	216 (1.34)
Urinary tract infection	178 (1.10)
Nasopharyngitis	163 (1.01)
Hypertension	134 (0.83)
Atrial fibrillation	121 (0.75)
Cerebrovascular accident	116 (0.72)
Osteoarthritis	114 (0.71)
Constipation	100 (0.62)
Myocardial infarction	91 (0.56)
Angina pectoris	84 (0.52)
Arthralgia	92 (0.57)
Anemia	83 (0.51)

These are cumulative data for nAMD patients who have completed observational periods of 1 year, 2 years, 3 years, and 4 years in LUMINOUS.

^†^Primary treated eye, ocular and non-ocular AEs ≥0.5%, for the total nAMD patients are shown.

The 95% confidence interval values were calculated only for AEs related to identified and potential risks that included: IOP increased (1.34, 1.73), hypertension (0.70, 0.98), cerebrovascular accident (0.59, 0.86), and myocardial infarction (0.45, 0.69).

AEs, adverse event (a patient with multiple occurrences of an AE is counted once per preferred term);

N, total number of patients; n, number of patients; nAMD, neovascular age-related macular degeneration.

Non-ocular AEs were reported in 23.02% (n = 3721) of patients in the safety set; the most common were fall (1.79%; n = 290), pneumonia, and lower respiratory tract infection (each, 1.34%; n = 216; Table 2). The incidence of non-ocular AEs suspected to be related to ocular injection was 0.46% (n = 74), of which 1.07% (n = 173), were reported to be related to ranibizumab alone. In all, 6.36% (n = 1028), 6.77% (n = 1095), and 9.88% (n = 1598) of patients had mild, moderate, and severe non-ocular AEs, respectively.

The incidence of ocular SAEs was 0.84% (n = 136) in the primary treated eye set; the most common ocular SAE was endophthalmitis reported in 0.14% (n = 23) of prior ranibizumab-treated patients with nAMD. Other most common ocular SAEs were retinal hemorrhage, (0.11%; n = 17), retinal detachment (0.09%; n = 14), cataract, and vitreous hemorrhage (both 0.07%; n = 12; Table 3). Two patients reported increased IOP and one patient reported uveitis. Ocular SAEs leading to discontinuation of ranibizumab treatment were reported in 0.11% (n = 18) of patients. The 95% CI values were calculated only for AEs and SAEs that were qualified as those with identified and potential risks. These included IOP increased, hypertension, cerebrovascular accident, and myocardial infarction for AEs and endophthalmitis, vitreous hemorrhage, retinal pigment epithelial tear, glaucoma, open angle glaucoma, cerebrovascular accident and myocardial infarction for SAEs. More details are provided in Tables 2 and 3 respectively.

**Table 3:** Ocular and non-ocular SAEs in prior ranibizumab-treated patients with nAMD.

Prior ranibizumab-treated group (N = 16,167)	
Preferred term	n, (%)
**Ocular SAEs**^†^	
Total	136 (0.84)
Endophthalmitis	23 (0.14)
Retinal hemorrhage	17 (0.11)
Cataract	12 (0.07)
Retinal detachment	14 (0.09)
Vitreous hemorrhage	12 (0.07)
Blindness	7 (0.04)
Retinal pigment epithelial tear	6 (0.04)
Glaucoma	6 (0.04)
Open angle glaucoma	5 (0.03)
Visual acuity reduced	5 (0.03)
Visual impairment	5 (0.03)
**Non-ocular SAEs**^†^	
Total	2208 (13.66)
Death	218 (1.35)
Pneumonia	194 (1.20)
Cerebrovascular accident	113 (0.70)
Fall	111 (0.69)
Myocardial infarction	91 (0.56)

These are cumulative data for nAMD patients who have completed observational periods of 1 year, 2 years, 3 years, and 4 years in LUMINOUS.

^†^Primary treated eye, ocular SAEs ≥5 events, non-ocular SAEs ≥0.5%, for the total nAMD patients are shown.

The 95% confidence interval values were calculated only for SAEs related to identified and potential risks that included: endophthalmitis (0.09, 0.21), vitreous hemorrhage (0.04, 0.13), retinal pigment epithelial tear (0.01, 0.08), glaucoma (0.01, 0.08), open angle glaucoma (0.01, 0.07), cerebrovascular accident (0.58, 0.84), myocardial infarction (0.45, 0.69).

N, total number of patients; n, number of patients; nAMD, neovascular age-related macular degeneration; SAEs, serious adverse events (a patient with multiple occurrences of an SAE is counted once per preferred term).

The incidence of non-ocular SAEs was 13.66% (n = 2208) in the safety set; pneumonia (1.20%; n = 194) and cerebrovascular accidents (0.70%; n = 113) were the most common non-ocular SAEs followed by myocardial infarction (0.64%; n = 16; Table 3). Non-ocular SAEs leading to discontinuation of ranibizumab treatment were reported in 4.71% (n = 762) of patients. Death was reported in 1.35% (n = 218) of patients. No new safety signals in addition to the well-characterized safety profile of ranibizumab were identified in this population.

## Discussion

LUMINOUS was the largest 5-year, prospective, observational, multicenter, open-label, single-arm, global study in medical retina that enrolled 30,138 patients across 42 countries to assess the effectiveness and safety of ranibizumab for all approved indications. To our knowledge, this present study is the first to report treatment patterns in prior ranibizumab-treated nAMD patients with a large patient population in a real-world setting. The results from the present analysis of prior ranibizumab-treated (<1 year) patients with nAMD demonstrate the continued effectiveness of ranibizumab during the first year of treatment in these patients. At Year 1, the VA achieved by prior ranibizumab-treated patients was comparable (54.7–57.8) irrespective of their duration of prior treatment. The mean number of injections over 1 year was 4.7. Around 40.3% (n = 1657) of prior ranibizumab-treated patients gained >0 to ≥15 letters from baseline at Year 1. Patients showed a trend of gaining VA when they received >6 injections. Overall, at the end of 1 year, vision was maintained in almost half (47.1%) of patients who were prior ranibizumab-treated for ≥5 years.

The results of the pivotal Phase III trials for ranibizumab, ANCHOR (11.3 letters) and MARINA (7.2 letters) showed that baseline VA was improved by 1–2 lines with monthly treatment [6, 8]. HORIZON, the extension study of ANCHOR, MARINA, and FOCUS trials, demonstrated a mean VA change from baseline VA of −4.1 letters at Year 1, in patients who received monthly ranibizumab treatment in the initial trials [9]. In contrast, the UK AMD EMR USERS GROUP (UK EMR study) observed a significant (globally adjusted p value, p<0.001) mean VA change of +2 letters (baseline of 58 letters) at 52 weeks [19, 20]. When nAMD patients were followed for a longer period in the SEVEN UP (~7 years) and the Comparison of Age-related Macular Degeneration Treatments Trials (CATT, 5 years) studies, a decline from baseline VA was observed [7, 21]. In the SEVEN UP study, the overall mean change in VA was −8.6 letters (from baseline entry into ANCHOR and MARINA) and −6.9 letters from HORIZON Month 24 [7]. In the CATT follow-up study, patients lost −10.8 (18.9) letters, over 3 years from the time of exit from the trial protocol [21]. The Fight Retinal Blindness (FRB) study reported a loss of 2.6 letters in 7 years in their patient population [5]. In the current LUMINOUS study, although overall vision was maintained, a small variation was observed in the mean VA change when presented by the patients’ duration of prior treatment. This variability could be due to differences in treatment patterns, the retreatment criteria, the different patient population and/or disease characteristics [5, 7, 21]. In addition, studies have observed that nAMD patients with a lower baseline VA achieve higher VA gains [5, 19]. Baseline VA and the mean age of the present study population are comparable with other real-world studies [5, 9, 19, 21]. The differences in the visual outcomes could be attributed to the observation that nAMD patients with similar baseline characteristics could respond differently to ranibizumab therapy [7]. It is important to consider that the patient population enrolled in other follow-up studies of RCTs followed strict inclusion/exclusion criteria and treatment regimen. The LUMINOUS study population is heterogeneous where treatment was at investigator’s discretion, and no loading dose was administered.

Overall, the treatment burden usually observed in real-world studies, including LUMINOUS, is low on average when compared with RCTs [5, 9, 19, 21]. In the current LUMINOUS study, we noted that patients who received >6 injections showed a trend towards gaining VA when compared with those who received <6 injections. Also, vision was maintained in patients who received prior ranibizumab treatment for >5 years before entering the LUMINOUS study. These data suggest that ranibizumab injection frequency may be associated with visual outcomes. The FRB study reported that in eyes with better baseline VA the net gain is much lower, but patients were able to maintain better levels of vision, and continued treatment due to the benefit of long-term outcomes [5]. These observations underscore the importance of patient management in terms of adequate follow-up and optimum number of injections to achieve better VA outcomes.

The prior ranibizumab-treated patients with nAMD from LUMINOUS were treated in real-world clinical practice settings across various countries with different healthcare systems. Among the 10 countries enrolling most of the prior ranibizumab-treated patients with nAMD, the mean number of injections up to 1-year ranged from 1.9 to 7.5. Most letters (−4.9) were lost in patients from Poland, which could be due to the low mean number of injections (3.1). Australia and Canada reported the highest number of injections (7.5), followed by the UK and Germany with 4.5 and 4.6 injections, respectively. The variation in the treatment exposure could be due to the treatment regimens followed in the countries; a PRN regimen in UK (patients recruited from the year 2011) and Germany (patients recruited from 2012), and a T&E approach in Australia and Canada (patients recruited from 2011 in both the countries) [22–25]. The mean number of injections is usually reported to be higher with a T&E approach than a PRN regimen, which was also observed in the present study. Apart from the differences in the treatment protocol, other factors, such as healthcare systems, including reimbursement of treatment, limited medical insurance coverage, access to treatment, treatment cost, patient compliance and follow-up, clinician’s decision to treat in subsequent visits, differences in the patient population, ocular characteristics, and comorbidities, could also account for the treatment exposure and visual outcomes variations observed in patients. Collecting more accurate and structured data that account for these differences could help to identify and overcome the difficulties of patients’ access to the right treatment [17].

Overall, the frequency of ocular and non-ocular SAEs and AEs over 5 years was low among the prior ranibizumab-treated patients in the LUMINOUS study. Ocular SAEs and AEs leading to discontinuation of ranibizumab and ocular AEs related to ranibizumab and/or injection were low. Overall, the ocular AEs in LUMINOUS were consistent with those observed in the RCT populations and consistent with the well-established safety profile of ranibizumab [6, 8, 9, 26]. The rate of endophthalmitis observed in the LUMINOUS population was low (0.14%) and comparable with other real-world evidence studies [9, 26]. The incidence rates of glaucoma, uveitis, and intraocular pressure increase were also low when compared with other studies [6, 8, 9, 26]. A retrospective, pooled analysis of safety data from four European nAMD registries in the LUMINOUS study also exhibited a favorable 1-year safety profile for ranibizumab in routine clinical practice [22]. The low rate of ocular and non-ocular AEs in this study confirms the safety of ranibizumab over years of exposure and suggests that there is possibly no cumulative risk from previous exposure to ranibizumab in patients with nAMD receiving ranibizumab therapy.

The strengths of the LUMINOUS study are inclusion of a large number of patients with diverse demographics, baseline characteristics, and pre-treatment status, without a mandated visit schedule where patients are treated at the investigator’s discretion, thereby closely depicting the real-world setting. The data from this study for prior ranibizumab-treated patients add information on the need for further treatment in the longer term to achieve better visual outcome in routine clinical practice.

It is important to note that there may have been a possibility of treatment bias due to the investigator’s decision, patient’s access to treatment, local healthcare systems and reimbursement policies, the flexible inclusion criteria, and variable treatment schedules across regions. The imaging data were not analyzed; there was no differentiation with regard to choroidal neovascularization sub phenotype, uniform retreatment criteria and best-corrected visual acuity protocol. There was also a difference in the number of patients enrolled between the countries. These factors may present some variations in interpretation of the results of the study. These limitations however, are common to most real-world evidence studies. This paper reports only Year-1 data for the prior ranibizumab-treated patients and longer term data would be required to evaluate the outcomes from this population.

LUMINOUS is the largest observational study and was conducted to assess the effectiveness and safety of ranibizumab for all approved indications. The results of the prior ranibizumab-treated patients from this study may help to guide clinicians’ recommendations for an appropriate number of injections to achieve optimal visual outcomes in patients with nAMD. The 5-year data from this study will help us to understand the long-term visual outcomes of ranibizumab treatment in nAMD patients in routine clinical practice.

## Meeting presentations

Data from this study were presented at the 18^th^ European Society of Retina Specialists (EURETINA) Congress, Vienna, Austria, 20‒23 September, 2018; the American Academy of Ophthalmology (AAO) Congress (New Orleans, USA [11–14 November, 2017] and Chicago, USA [27–30 October, 2018).

## Supporting information

S1 Table List of Independent Ethics Committees (IEC) or Institutional Review Boards (IRB) by study center. (DOCX)

S2 Table Baseline demographics and ocular characteristics of the top 10 countries, which enrolled most prior ranibizumab-treated patients. n, number of patients; CRT, central retinal thickness; IOP, intraocular pressure; SD, standard deviation; UK, the United Kingdom.
(DOCX)

S3 Table VA outcomes and treatment exposure of top 10 countries, which enrolled most prior ranibizumab-treated patients. SD, standard deviation; UK, the United Kingdom; VA, visual acuity.
(DOCX)

S4 Table List of principal investigators on the LUMINOUS study. (DOCX)
