# Supplementary material for: Ranibizumab treatment patterns in prior ranibizumab-treated neovascular age-related macular degeneration patients: Real-world outcomes from the LUMINOUS study
Source: PLoS One. 2020 Dec 30;15(12):e0244183. doi: 10.1371/journal.pone.0244183 (PMC7773197; doi:10.1371/journal.pone.0244183)
Supplement: S2 Table — n, number of patients; CRT, central retinal thickness; IOP, intraocular pressure; SD, standard deviation; UK, the United Kingdom. (DOCX) [file pone.0244183.s002.docx]

**S2 Table**. Baseline demographics and ocular characteristics of the top 10 countries, which enrolled most prior ranibizumab-treated patients

| **Country** | **Mean (SD) age in years** | **Females,  n (%)** | **Median time since diagnosis (days)** | **Mean (SD) IOP (mmHg)** | **Mean (SD) CRT**  **(µm)** |
| --- | --- | --- | --- | --- | --- |
| Australia | 79.9 (8.1) | 66.4 | 821 | 14.3 (3.8) | 252.2 (71.1) |
| Canada | 79.6 (8.6) | 61.3 | 618.5 | 16.3 (4.0) | 264.2 (86.3) |
| China | 70.7 (9.9) | 45.5 | 452 | 13.5 (2.9) | 325.6 (148.8) |
| France | 80.6 (7.2) | 71.1 | 864 | 15.0 (3.6) | 280.9 (90.5) |
| Germany | 78.5 (8.7) | 64.6 | 741 | 15.2 (3.4) | 317.8 (108.2) |
| Hungary | 74.6 (7.9) | 63.7 | 421 | 14.8 (2.5) | 318.6 (125.0) |
| Japan | 77 (8.0) | 30.9 | 862 | 13.4 (3.3) | 277.1 (104.2) |
| Poland | 72.9 (8.7) | 66.8 | 640.5 | 15.7 (3.1) | 335.2 (139.4) |
| Portugal | 79 (8.1) | 59.5 | 927 | 17.2 (3.4) | 281.1 (82.9) |
| UK | 80.1 (8.0) | 63.7 | 501 | 16.0 (3.4) | 263.0 (85.9) |

n, number of patients

CRT, central retinal thickness; IOP, intraocular pressure; SD, standard deviation; UK, the United Kingdom
