# Supplementary material for: Ranibizumab treatment patterns in prior ranibizumab-treated neovascular age-related macular degeneration patients: Real-world outcomes from the LUMINOUS study
Source: PLoS One. 2020 Dec 30;15(12):e0244183. doi: 10.1371/journal.pone.0244183 (PMC7773197; doi:10.1371/journal.pone.0244183)
Supplement: S3 Table — SD, standard deviation; UK, the United Kingdom; VA, visual acuity. (DOCX) [file pone.0244183.s003.docx]

**S3 Table**. VA outcomes and treatment exposure of top 10 countries, which enrolled most prior ranibizumab-treated patients

| **Country** | **Enrolled patients,**  **(n)** | **Patients with evaluable baseline and 1-year VA, (n)** | **Baseline VA, mean (SD)** | **Month 12 VA,**  **mean (SD)** | **Change in VA at 1-year,**  **mean (SD)** | **Mean number of injections at  1-year, mean (SD)** |
| --- | --- | --- | --- | --- | --- | --- |
| Australia | 1531 | 978 | 62.4 (17.3) | 61.3 (18.2) | −1.2 (11.0) | 7.5 (2.7) |
| Canada | 1701 | 1158 | 52.5 (23.6) | 51.8 (24.3) | −0.7 (14.2) | 7.5 (2.8) |
| China | 684 | 99 | 47.6 (18.9) | 45.4 (21.8) | −2.2 (18.1) | 2.2 (1.8) |
| France | 598 | 277 | 62.6 (17.6) | 60.5 (20.8) | −2.1 (13.6) | 4.0 (2.4) |
| Germany | 271 | 158 | 56.7 (19.1) | 55.4 (21.2) | −1.3 (12.8) | 4.6 (2.4) |
| Hungary | 343 | 245 | 60.4 (14.8) | 56.6 (16.9) | −3.8 (10.9) | 2.9 (2.3) |
| Japan | 1231 | 738 | 62.7 (20.9) | 62.0 (22.0) | −0.7 (12.3) | 3.4 (2.6) |
| Poland | 343 | 208 | 53.4 (19.6) | 53.4 (19.4) | −0.1 (13.4) | 3.1 (2.4) |
| Portugal | 327 | 173 | 52.7 (19.4) | 47.8 (19.7) | −4.9 (12.0) | 1.9 (1.4) |
| UK | 7030 | 4683 | 58.6 (16.9) | 56.6 (19.1) | −2.0 (11.6) | 4.5 (3.0) |

n, number of patients

SD, standard deviation; UK, United Kingdom; VA, visual acuity
